# Validation of Recombinant Salivary Protein PpSP32 as a Suitable Marker of Human Exposure to *Phlebotomus papatasi*, the Vector of *Leishmania major* in Tunisia

**DOI:** 10.1371/journal.pntd.0003991

**Published:** 2015-09-14

**Authors:** Soumaya Marzouki, Wafa Kammoun-Rebai, Jihene Bettaieb, Maha Abdeladhim, Saoussen Hadj Kacem, Rania Abdelkader, Sami Gritli, Jomaa Chemkhi, Hamide Aslan, Shaden Kamhawi, Afif Ben Salah, Hechmi Louzir, Jesus G. Valenzuela, Melika Ben Ahmed

**Affiliations:** 1 Laboratory of Transmission, Control and Immunobiology of Infection, LR11IPT02, Institut Pasteur de Tunis, Tunis, Tunisia; 2 Laboratory of Medical Parasitology, Biotechnologies and Biomolecules, LR11IPT06, Institut Pasteur de Tunis, Tunis, Tunisia; 3 Faculté de Médecine de Tunis, Université Tunis El Manar, Tunis, Tunisia; 4 Vector Molecular Biology Section, Laboratory of Malaria and Vector Research, National Institute of Allergy and Infectious Diseases, National Institutes of Health, Rockville, Maryland, United States of America; 5 Department of Pathology, Charles Nicolle Hospital, Tunis, Tunisia; 6 Laboratory of Molecular Epidemiology and Experimental Pathology, LR11IPT04, Institut Pasteur de Tunis, Tunis, Tunisia; Lancaster University, UNITED KINGDOM

## Abstract

**Background:**

During a blood meal, female sand flies, vectors of *Leishmania* parasites, inject saliva into the host skin. Sand fly saliva is composed of a large variety of components that exert different pharmacological activities facilitating the acquisition of blood by the insect. Importantly, proteins present in saliva are able to elicit the production of specific anti-saliva antibodies, which can be used as markers for exposure to vector bites. Serological tests using total sand fly salivary gland extracts are challenging due to the difficulty of obtaining reproducible salivary gland preparations. Previously, we demonstrated that PpSP32 is the immunodominant salivary antigen in humans exposed to *Phlebotomus papatasi* bites and established that humans exposed to *P*. *perniciosus* bites do not recognize it.

**Methodology/Principal Findings:**

Herein, we have validated, in a large cohort of 522 individuals, the use of the *Phlebotomus papatasi* recombinant salivary protein PpSP32 (rPpSP32) as an alternative method for testing exposure to the bite of this sand fly. We also demonstrated that screening for total anti-rPpSP32 IgG antibodies is sufficient, being comparable in efficacy to the screening for IgG2, IgG4 and IgE antibodies against rPpSP32. Additionally, sera obtained from dogs immunized with saliva of *P*. *perniciosus*, a sympatric and widely distributed sand fly in Tunisia, did not recognize rPpSP32 demonstrating its suitability as a marker of exposure to *P*. *papatasi* saliva.

**Conclusions/Significance:**

Our data indicate that rPpSP32 constitutes a useful epidemiological tool to monitor the spatial distribution of *P*. *papatasi* in a particular region, to direct control measures against zoonotic cutaneous leishmaniasis, to assess the efficiency of vector control interventions and perhaps to assess the risk of contracting the disease.

## Author Summary

Leishmaniasis results from an infection by *Leishmania* parasites that are transmitted through the bites of infected sand flies. This disease affects millions of people worldwide. Zoonotic cutaneous leishmaniasis is widespread in Central Tunisia and constitutes an actual public health problem. *Leishmania major*, the etiological agent, is transmitted by the sand fly vector *Phlebotomus papatasi*. Saliva of sand flies contains several pharmacologically active components that play a key role in the acquisition of the blood meal and the establishment of the parasites, thus enhancing the infection. Some of these molecules are able to elicit the production of specific antibodies, which can be used as markers of exposure to the vector’s bite. Herein, using a large cohort of individuals, we have validated the use of *P*. *papatasi* recombinant salivary protein PpSP32 (rPpSP32) as an alternative method to standard entomological studies for testing exposure to the bite of this sand fly in humans. rPpSP32 represents a promising epidemiological tool to monitor the spatial distribution of *P*. *papatasi*, direct control measures against zoonotic cutaneous leishmaniasis, evaluate the efficiency of vector control interventions and potentially assess the risk of contracting the disease.

## Introduction

Leishmaniasis affects millions of people worldwide. It is a heterogeneous group of diseases caused by *Leishmania* parasites. Zoonotic cutaneous leishmaniasis (ZCL) is the most prevalent form in North Africa and is widespread in Central Tunisia. With an annual incidence of ~5,000 cases, it constitutes an actual public health problem [1, 2]. *Leishmania major*, an Old World *Leishmania* species is the etiological agent which is transmitted by the sand fly vector, *Phlebotomus (P*.*) papatasi* [3].

As they bite mammalian host skin, phlebotomine sand flies inject a range of salivary molecules that facilitate blood meal acquisition [4]. Moreover, the co-inoculation of *Leishmania* parasites with saliva during an infected bite enhances disease progression through the action of immunomodulatory molecules [5–7].

Humans and animals exposed to sand fly bites or experimentally immunized with saliva develop antibodies that specifically target most sand fly salivary proteins [8–17]. The level of anti-saliva antibodies has been frequently correlated not only to the number of bites received [9–11, 15] but also to the risk of acquiring leishmaniasis, particularly cutaneous forms of the disease [16–17]. Thus, antibodies directed against sand fly saliva have been proposed as useful epidemiological markers of vector exposure in leishmaniasis endemic areas [12, 13, 18–20]. Serological tests using total sand fly salivary gland extracts to assess vector exposure are challenging due to the difficulty of obtaining large and reproducible salivary gland preparations. Another limitation is the potential lack of specificity. Antibodies generated against saliva of a particular species may cross react with salivary protein homologues present in different species [21–22]. Thus, the use of recombinant proteins exhibiting predominant species-specificity may overcome such issues [11, 12, 19, 23, 24].

We have recently identified PpSP32 as the immunodominant salivary protein from *P*. *papatasi* saliva as this protein is targeted by antibodies in the majority of people living in an endemic area of ZCL in Tunisia. We have also established that humans bitten by *P*. *perniciosus*, the vector of *Leishmania infantum (L*. *infantum)* in Tunisia do not recognize PpSP32 although this species is abundant in areas where *P*. *papatasi* is prevalent. Moreover, we have demonstrated the suitability of using the recombinant form of this protein in a serological test [24].

Herein, we aimed to validate the use of the recombinant salivary protein PpSP32 (rPpSP32) as a suitable marker of human exposure to *P*. *papatasi* bites in a large cohort of 522 individuals. Altogether, our results substantiate the use of this protein as a promising epidemiological tool for accurate monitoring of exposure to *P*. *papatasi*, the vector of ZCL in Tunisia.

## Methods

### Ethics statement

The Ethics Committee of the Pasteur Institute of Tunis approved the study (protocol number 07–0018). For the collection of blood samples and subsequent analyses, parents/guardians provided written informed consent on behalf of all child participants.

All animal procedures were reviewed and approved by the National Institute of Allergy and Infectious Diseases (NIAID) Animal Care and Use Committee under Animal protocol LMVR7E and handled in accordance to the Guide for the Care and Use of Laboratory Animals and with the NIH OACU ARAC guidelines.

### Salivary glands from Tunisian *Phlebotomus papatasi* and *Phlebotomus perniciosus*

*Phlebotomus papatasi* salivary glands were obtained from colonized 3 to 5 days old females originating from an endemic focus of ZCL, El Felta, located in Sidi Bouzid Governorate in Central Tunisia (North Africa) as previously described [24, 25]. *Phlebotomus perniciosus* (200 flies) were collected from an endemic area of visceral leishmaniasis in Zaafaran, a city in the Kef Governorate (North Western Tunisia). Sand flies were captured using CDC miniature light traps placed in animal shelters. Unfed *P*. *perniciosus* females were dissected to provide salivary glands. The unfed status was based on the absence of blood meal.

For salivary glands dissection, the head was cleanly severedfrom the thorax by crossing two fine entomological pins. The salivary glands, visible astwo oval luminescent lobes attached to the head, were released and transferred to a tube containing phosphate buffered saline (PBS), then disrupted by 3 freezing and thawing cycles as previously described [24]. The supernatants were stored in 10% glycerol at a concentration of 200 glands/ml at -80°C until use.

### Study population and samples

Peripheral blood samples were collected from 522 children (age range 7–21 years, median 13 years) during a cross sectional household survey carried out between January and May 2009. The donors were living in five localities in Central Tunisia endemic for ZCL caused by *L*. *major* and characterized by the abundance of *P*. *papatasi* [26]. The localities, as described by Bettaieb et al. [27] share the same topography and climate and are located in two adjacent governorates, Sidi Bouzid and Kairouan in the arid zone of Tunisia. One hundred and thirty three children were from Mnara, 134 from Ksour, 104 from Dhouibet, 74 from Mbarkia and 77 from Msaadia. All donors were part of the Tropical Medicine Research Centers (TMRC) study entitled “Key Determinants of the Natural History Of *Leishmania major* Infection”. Details of the endemic areas and several clinical and biological parameters pertaining to the donors such as the presence of typical scars or anti-*Leishmania* delayed type hypersensitivity skin test have been collected.

### Study design

**Fig 1:**
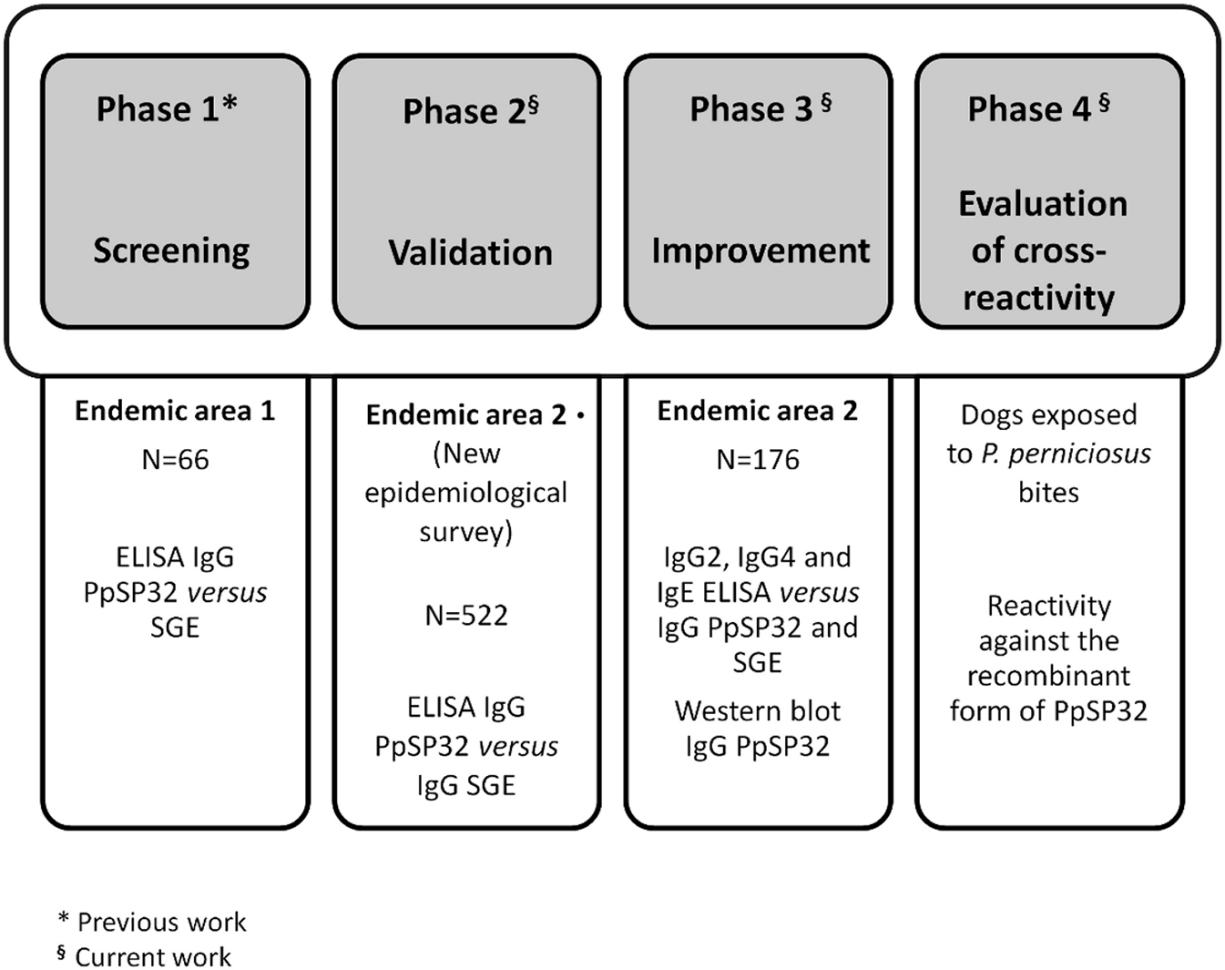
Study design. Our study was divided in four phases. The first phase, an ELISA test previously performed on 66 serum samples from children living in central regions of Tunisia (endemic area 1), indicated that rPpSP32 may be a suitable marker of exposure to *Phlebotomus papatasi* bites [12]. The second phase consisted of the validation of this serological test as a marker of vector exposure in a larger cohort in a new population (522 donors from endemic area 2). The third phase of the study consisted of improvement of the performance of the serological test by assessing the anti-rPpSP32 IgG subclasses (IgG4 and IgG2) and IgE antibody response by ELISA or the presence of IgG anti-rPpSP32 by Western-blot. This phase was performed on 176 representative sera selected from the 522 new donors. In the last phase of the study, the specificity of the rPpSP32 serological test was assessed by testing the reactivity of sera obtained from dogs exposed to bites of *P*. *perniciosus bites* against either rPpSP32 or *P*. *papatasi* salivary gland extract.

The study design consisted of four phases **(Fig 1).**

The first phase has been previously performed [24] on 66 serum samples obtained from children living in two localities (El Guettar and Souk Ejdid) in Central Tunisia corresponding to the endemic area 1. It was shown that rPpSP32 was strongly recognized by people naturally exposed to *P*. *papatasi* bites suggesting the suitability of using this protein in a serological test to monitor exposure to *P*. *papatasi* bites.

Here, we completed the remaining three phases of the study. The second phase of the study consisted of the validation of this serological test as a marker of vector exposure in a new population survey (Endemic area 2) using the Receiver-Operator Characteristic (ROC) curves and serology against total salivary gland extract (SGE) as the gold standard. In fact, the serum samples obtained from 522 donors living in the five localities detailed above have been tested against SGE of *P*. *papatasi* and 209 were positive while 313 were negative. The cut-off value of the anti-SGE enzyme-linked immunosorbent assay (ELISA) was 0.404 and was established as previously described [17]. The ROC curves have been used to identify the effectiveness of rPpSP32 for the identification of anti-SGE antibody positivity. They led us to determine cut-offs combining highest sensitivity and specificity for this discrimination.

The third phase of the study, undertaken on a set of serum samples obtained from donors of the endemic area 2, investigated whether the performance of the serological test can be improved by assessing the anti-rPpSP32 antibody response in different IgG subclasses (IgG4, IgG2) and IgE by ELISA or the presence of IgG anti-rPpSP32 by Western-blot. For comparison of the performance of antibody subclasses and IgE using an anti-rPpSP32 ELISA, 176 representative sera were selected from the 522 initial samples. In this representative subgroup, positive sera (107 samples) were selected so that they cover the different range of positivity found within the initial samples.

In the final phase of the study, we tested the specificity of rPpSP32 to saliva of *P*. *papatasi* by analyzing the reactivity of sera from dogs immunized with bites of *P*. *perniciosus*, the vector of visceral leishmaniasis due to *L*. *infantum*, a species that occurs sympatrically with *P*. *papatasi* in parts of Tunisia.

### Detection of human IgG anti-SGE and anti-rPpSP32 antibody

Specific IgG anti-saliva and anti-rPpSP32 antibodies were measured by ELISA as previously described [17, 24]. Briefly, the wells were coated overnight with SGE from *P*. *papatasi* or *P*. *perniciosus* (0.5 glands per well = 0.25μg/well) or rPpSP32 (2μg/ml = 0.1μg/well) in 0.1M carbonate-bicarbonate buffer (pH 9.6) at 4°C. After washing and blocking free binding sites for 1 hour at 37°C with PBS-Tween-0.5% gelatin, sera diluted at 1:200 were incubated for 2 hours at 37°C. After washing, peroxidase-conjugated anti-human IgG antibody (Sigma, St. Louis, MO) was incubated for 1 hour at 37°C. Tetramethylbenzidine (TMB) (BD Biosciences, San Diego, CA) was then used to visualize antibody-antigen complexes. The absorbance was measured at 450 nm wavelength using an automated ELISA reader (Awareness Technology Inc, Palm City, FL).

### Detection of human IgG subclasses (IgG2, IgG4) and IgE anti-rPpSP32 antibodies by ELISA

Specific IgG2, IgG4 and IgE antibodies against rPpSP32 were measured using ELISA. The optimal conditions of antigen concentration as well as sample, primary antibody and streptavidine-horseradish peroxidase dilutions were previously determined [17, 24]. The wells coated with rPpSP32 were incubated with diluted serum samples (1:200) for 2 hours at room temperature. After 6 washes, biotin-conjugated anti-human IgG isotypes (Sigma) or IgE (BD Biosciences) were incubated at 37°C for 1 hour at a dilution of 1:20,000 for IgG2, IgG4 and 1:250 for IgE. After 8 additional washes, streptavidin-horseradish peroxidase diluted at 1:15,000 (Amersham, Little Chalfont Buckinghamshire, UK) was added for 30 minutes at 37°C. Antibody-antigen complexes were visualized using TMB (BD Biosciences). The absorbance was measured by an automated ELISA reader (Awareness Technology Inc.) at 450nm. The cut-off for the assays was the mean OD of negative controls plus three standard deviations.

### Western blot

Recombinant PpSP32 (0.7μg per well) was run on a 15% sodium dodecyl sulfate- polyacrylamide gel electrophoresis (SDS-PAGE). The optimal conditions of antigen concentration as well as sample, primary antibody and streptavidine-horseradish peroxidase dilutions were previously determined [24]. After separation the proteins were transferred onto a nitrocellulose membrane, incubated for 2 hours with blocking buffer containing 5% non-fat milk and then cut into strips. Each strip was then incubated overnight with human sera diluted at 1:200. After washing, horseradish peroxidase-linked anti-human IgG antibody (Sigma) was incubated for 1 hour at room temperature. Positive bands were visualized using enhanced chemiluminescence (Amersham, Saclay, France).

### Exposure of dogs to *P*. *perniciosus* bites

Four month-old male Beagles were purchased from Marshall Farms. Dogs were experimentally exposed to the bites of 20 *P*. *perniciosus* females on the shaved neck four times every two weeks using custom-made feeders. On average, the percent of fed flies per animal was 44%. Dogs were bled prior to and after the last exposure to bites. *P*. *perniciosus* flies used for these experiments were obtained from a colony that originated from Italy which is currently maintained at Walter Reed Army Institute of Research (WRAIR). This procedure was previously used in animals exposed to *Lutzomyia longipalpis* bites to successfully develop antibodies against sand fly salivary proteins in dogs [28].

Dog sera, obtained two weeks after the last immunization, were tested for IgG antibodies to *P*. *perniciosus* saliva, *P*. *papatasi* saliva and to rPpSP32 using ELISA as described above.

### Statistical analysis

Spearman’s rank correlation was used to assess the correlation between the OD of tested sera obtained by ELISA against recombinant PpSP32 and total SGE. For continuous variables, the Mann-Whitney U test was used. Receiver operating characteristic (ROC) curves were used to evaluate the performance of the rPpSP32 as well as IgG subclasses and IgE in the determination of anti-SGE positivity. The optimal cut-off level identified from the ROC curve was used for calculation of sensitivity and specificity of SGE and rPpSP32 separately using standard methods. Statistical significance was assigned to a value of p < 0.05. All Statistical analyses and graphs were performed using GaphPad Prism v5.0 software.

## Results

**Fig 2:**
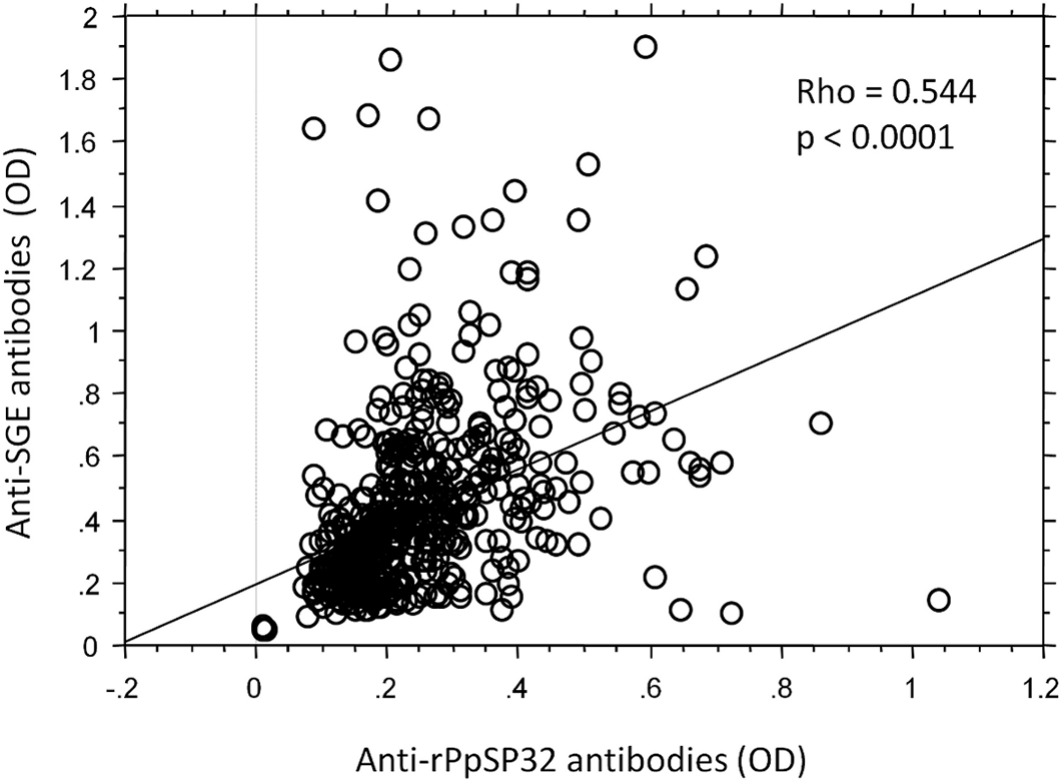
Correlation between antibody production against the total extract of saliva (SGE) and the recombinant form of SP32 (rPpSP32). Sera from five hundred and twenty two donors living in the endemic area 2 were included. The presence of IgG antibodies against SGE and rPpSP32 was tested by ELISA. Data were analyzed using Spearman test.

**Fig 3:**
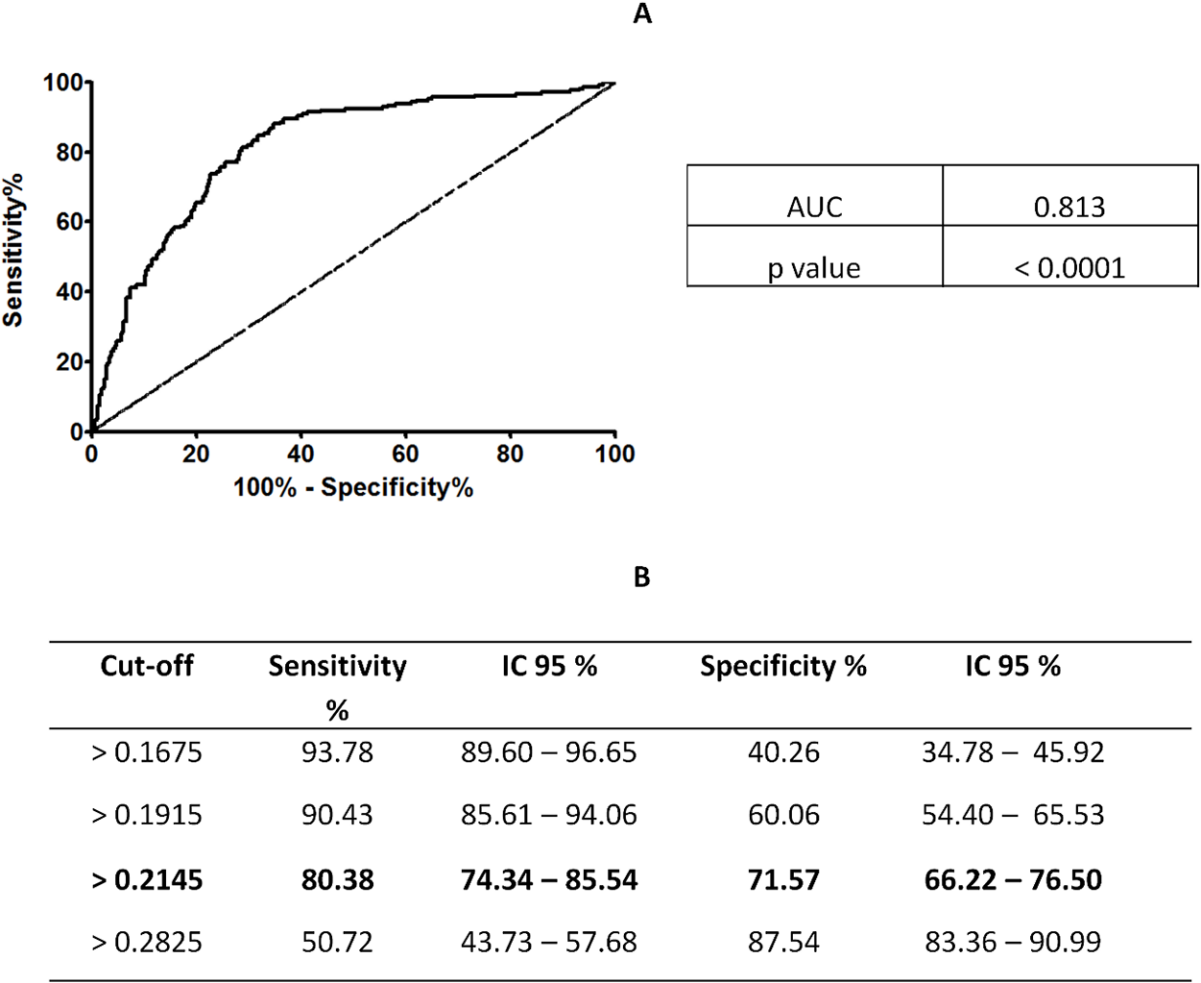
ROC curve of rPpSP32 antibodies predicting ELISA positivity against SGE. **(A)** ROC curve was performed using data of the serology against the rPpSP32 obtained from 209 individuals exhibited anti-SGE antibodies and 313 who did not. The area under curves (AUC) and the p value of the ROC curve are shown. **(B)** Sensitivity and specificity with 95% confidence interval (CI) for different cut-off values are also shown.

**Fig 4:**
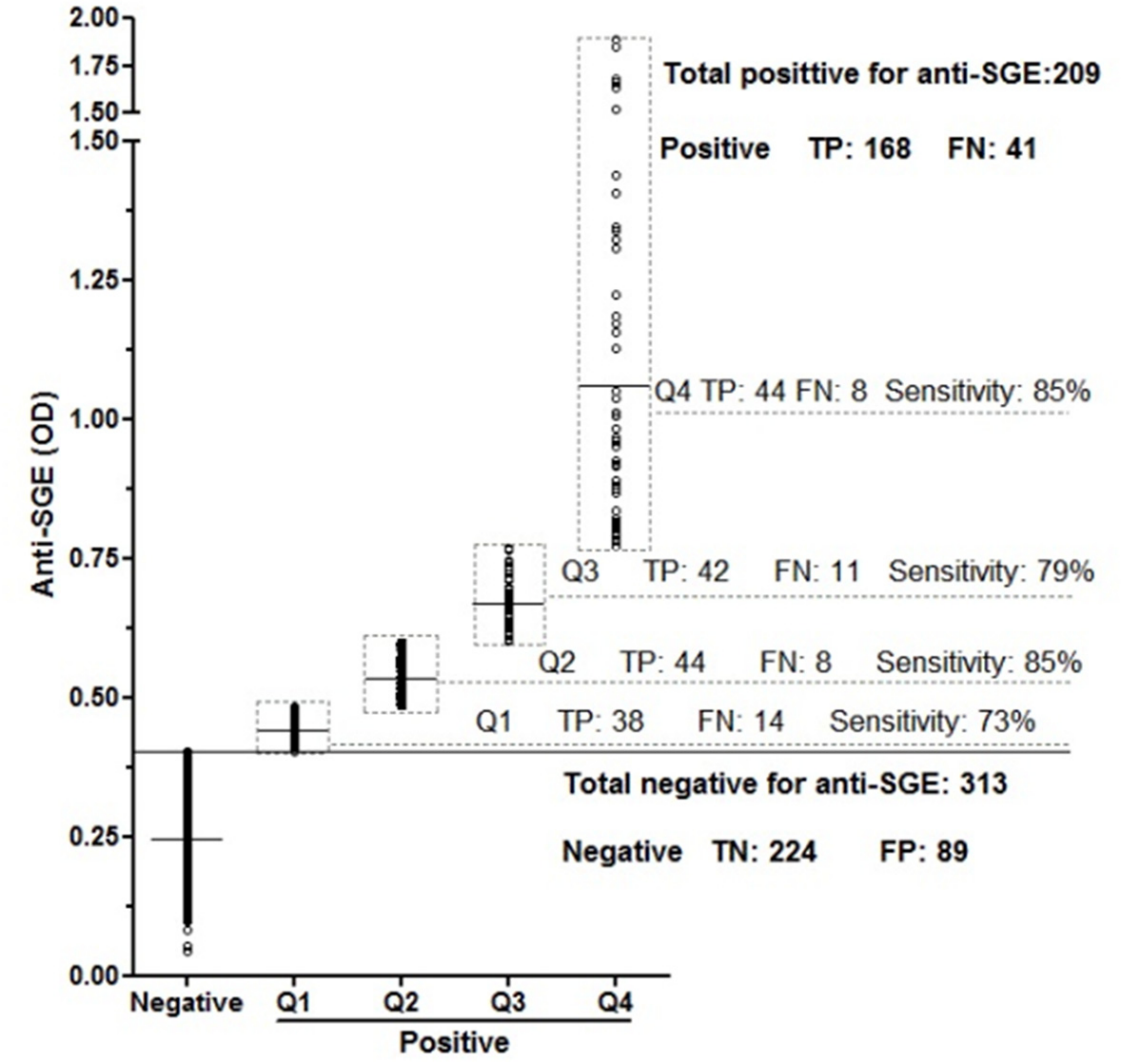
Validation of rPpSP32 as a marker of human exposure to *P*. *papatasi* saliva. A total of 522 individuals from endemic area 2 were tested for anti-SGE and anti-rPpSP32 antibodies. The anti-SGE antibody titers (OD) were grouped into quartiles (Q). For each quartile, the sensitivity, the number of true positives (TP) and false negatives (FN) are shown. For anti-SGE negative sera, the numbers of true negatives (TN) and false positives (FP) are indicated.

We have previously demonstrated that PpSP32 is the immunodominant target of the antibody response to *P*. *papatasi* saliva [24]. We have also shown that rPpSP32 behaves similarly to the native salivary protein and is likely to be a suitable marker of human exposure to *P*. *papatasi* bites and potentially for assessing the risk of developing the disease **(Phase 1, Fig 1)** [24]. Herein, in the second phase of the study, we have further assessed the effectiveness of rPpSP32 in determining anti-SGE antibodies using a larger cohort of people (n = 522) living in other endemic areas of ZCL in Tunisia where *P*. *papatasi* is prevalent **(Fig 1)**. Such donors were previously tested for antibodies against *P*. *papatasi* SGE and 209 were positive while 313 were negative. Using rPpSP32 serology, 257 of the tested sera were positive while 265 were negative. A significant correlation was found between the OD obtained for all the tested donors with rPpSP32 and those obtained with the total extracts of salivary glands (p < 0.0001, r = 0.544) **(Fig 2)**. The effectiveness of rPpSP32 as a biomarker of *P*. *papatasi* sand fly exposure was estimated by the area under the ROC curve (AUC) established using the SGE ELISA test as a gold standard. Thus, the serology using rPpSP32 was able to distinguish individuals exposed to *P*. *papatasi* saliva (AUC: 0.813; p < 0.0001) **(Fig 3A)**. The overall performance of the test using the rPpSP32 was satisfactory with a sensitivity of 80.38% (95% CI: 74.34–85.54) and a specificity of 71.57% (95% CI: 66.22–76.50) when the cut-off value was fixed at 0.21 OD **(Fig 3B).** Interestingly, no correlation was found between the level of anti-PpSP32 antibodies and the age of the donors (p>0.05). In order to test the performance of the serology in donors with high and low antibody titers against SGE, we stratified SGE positive cases in quartiles according to optical density values and calculated discordant results for each quartile (**Fig 4**). No significant differences were noted in the effectiveness of prediction in individuals with high or low anti-SGE antibody titers **(Fig 4).**

**Fig 5:**
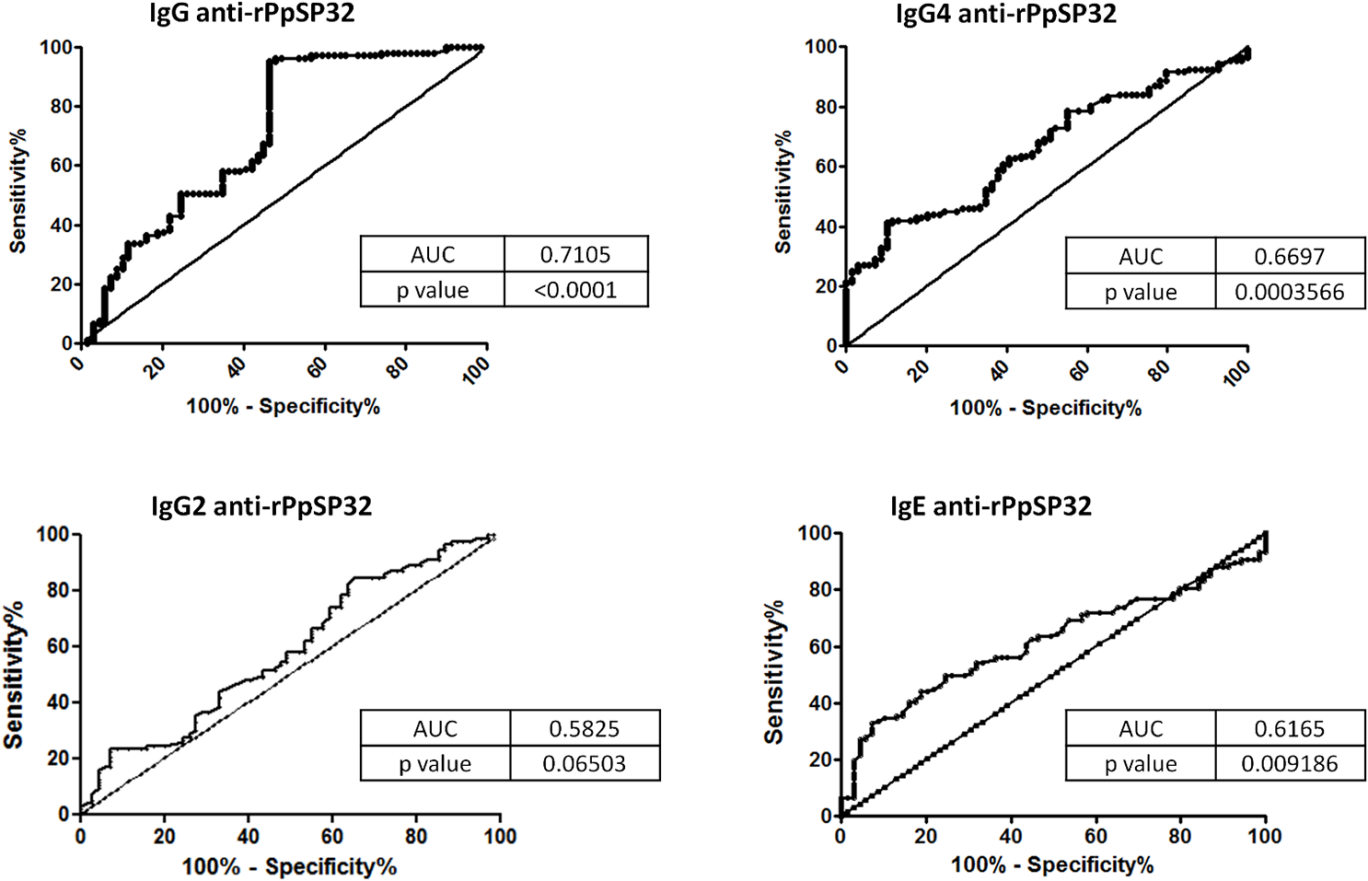
ROC curves predicting ELISA positivity against rPpSP32 for IgG, IgG subclasses and IgE. Levels of IgG, IgG2, IgG4 and IgE antibodies directed against *P*. *papatasi* SP32 were studied in serum samples of 176 representative participants. The area under curves (AUC) and the p value of the ROC curve are shown.

**Fig 6:**
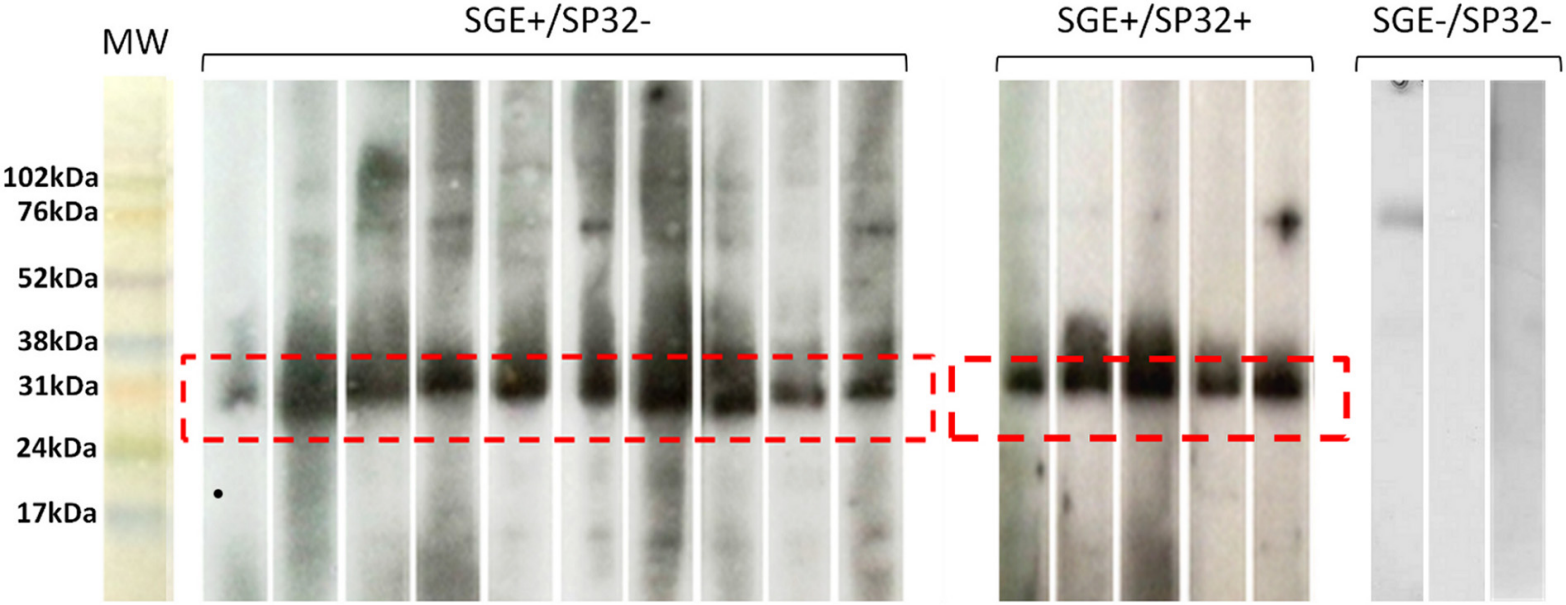
Western blot analysis of anti-rPpSP32 IgG antibodies. The recombinant form of PpSP32 was run on a 15% SDS-PAGE gel. Western blot analysis testing IgG antibodies against rPpSP32 was performed on three groups of randomly selected sera: 10 sera that were positive in an ELISA test for anti-SGE antibodies and negative for anti-SP32 antibodies (SGE+/SP32-), 20 concordant sera (SGE+/SP32+) and 10 negative samples (SGE-/SP32-). Representative sera are shown from each group. Dashed line delineates the bands corresponding to PpSP32. The RPN800E molecular weight (MW) marker (Amersham) was used.

To improve the performance of the test, the most frequent IgG subclasses (IgG2, IgG4) and IgE anti-SGE antibodies were investigated in the sera of a representative set of the endemic area 2 cohort (n = 176) **(Phase 3, Fig 1).** The overall performance of the IgG4 rPpSP32 ELISA test was higher than that of IgG2 or IgE rPpSP32 yet lower compared to the performance of the total IgG rPpSP32 ELISA (AUC 0.6697 *versus* 0.7105 and a p = 0.0003566 *versus* p < 0.0001) **(Fig 5).** A significant correlation was found between the OD obtained with IgG rPpSP32 for all donors and those obtained with IgG4 rPpSP32 (p = 0.0007, r = 0.256), IgG2 rPpSP32 (p = 0.0045, r = 0.215), and IgE rPpSP32 (p = 0.0057, r = 0.209). As IgG rPpSP32 serology was the most suitable test for use as a marker of *P*. *papatasi* exposure, we tested whether we would increase the performance of the serology using Western blot. Ten serum samples of donors that exhibited IgG anti-SGE antibodies but that were negative with IgG rPpSP32 ELISA were tested by Western blot. All sera demonstrated the presence of antibodies against salivary rPpSP32 protein, thus leading to the recovery of false negatives obtained with the IgG rPpSP32 ELISA **(Fig 6).** Western blot performed on twenty randomly selected concordant sera that exhibited SGE+/rPpSP32+ by ELISA showed a similar response confirming the presence of antibodies to rPpSP32 in all the tested positive sera **(Fig 6).**

**Fig 7:**
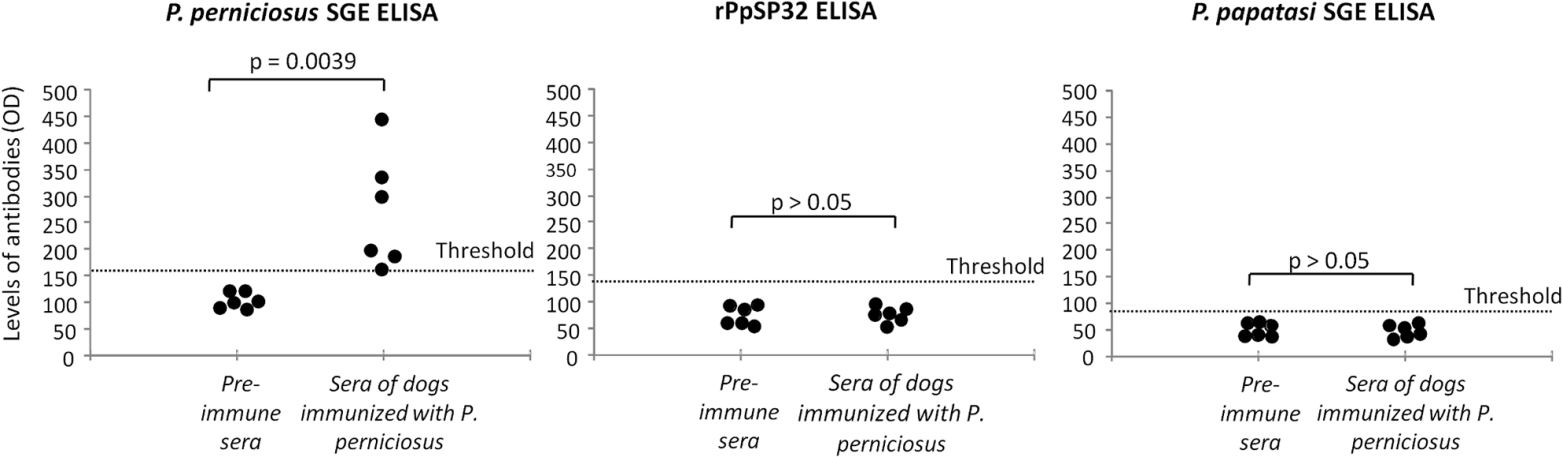
Analysis of cross-reactivity between *P*. *papatasi* SP32 and *P*. *perniciosus* saliva. The reactivity of immune and pre-immune sera obtained from dogs exposed to *P*. *perniciosus* bites was tested by ELISA against *P*. *perniciosus* salivary gland extract (SGE), rPpSP32 or *P*. *papatasi* SGE. The threshold of positivity was calculated as the mean optical density (OD) of pre-immune sera plus 3 standard deviations.

Prior to validating rPpSP32 as a suitable marker of exposure to *P*. *papatasi*, we assessed the specificity of the test by evaluating sera of animals exposed to another sand fly species prevalent in Tunisia, *P*. *perniciosus* **(Phase 4, Fig 1)**. Indeed, PpSP32, the immunodominant target of *P*. *papatasi* saliva, has homologues in at least five other sand fly species (*P*. *argentipes*, *P*. *ariasi*, *P*. *perniciosus*, *P*. *sergenti* and *L*. *longipalpis*) [29, 30]. Among them, *P*. *perniciosus* is particularly prevalent in Tunisia while *P*. *sergenti* represents only 1% of sand fly species in semi arid areas [26]. Thus, we exposed dogs to bites of 20 *P*. *perniciosus* sand flies and tested the reactivity of their recovered sera against rPpSP32. Overall, 44% of sand flies bloodfed on each dog per exposure. As shown in **Fig 7**, sera obtained from dogs exposed to *P*. *perniciosus* bites recognized the salivary proteins present in *P*. *pernicosus* SGE but had no reactivity against rPpSP32 *or P*. *papatasi* SGE.

## Discussion

The use of insect salivary proteins has become an attractive alternative to measure the exposure to vectors in humans and animals reservoirs [8, 31, 32]. Salivary proteins of mosquitoes [33], tsetse flies [34], and triatomine bugs [35] have been successfully used as biomarkers for vector exposure. For sand flies, the identification of salivary proteins targeted by the humoral response of humans could help in developing potential epidemiological markers of sand fly exposure in endemic areas of leishmaniasis [18, 23]. Indeed, monitoring of human antibodies directed against sand fly saliva may constitute a useful indicator of the spatial distribution of sand flies in a particular region and be helpful in directing vector control measures. Serological tests using salivary antigens would, in fact, be more cost-effective to assess the effectiveness of anti-vector strategy than using entomological methods or measuring the incidence of vector-borne disease [36]. Furthermore, the presence of a correlation between anti-saliva antibodies and the risk of leishmaniasis underlies the importance of developing such tests for predicting the risk of leishmaniasis. However, large-scale serological studies using sand fly salivary gland extracts are limited by considerable variability in salivary gland stocks [37]. The use of recombinant sand fly salivary proteins represents an attractive alternative. Accordingly, the yellow proteins LJM11 and LJM17 have been validated as specific markers of exposure to *Lutzomyia (Lu*.*) longipalpis* saliva in humans [19, 23] while the apyrases rSP01B and rSP01 and the yellow protein rSP03B show promise as markers of canine exposure to *P*. *perniciosus* [8, 31].

PpSP32, the main target of the antibody response against saliva of *P*. *papatasi*, seems to represent a good candidate for assessing human exposure to vector bites. Indeed, we have previously suggested the effectiveness of the recombinant form of PpSP32 in predicting anti-SGE antibodies using a relatively small sample of individuals (n = 66), in which 42 were positive and 24 were negative for anti-SGE [24]. Herein, by testing a larger cohort of donors (n = 522) living in different endemic areas of ZCL, we confirmed the positive correlation between the results obtained using SGE and those obtained using rPpSP32. As attested by the ROC curve, the overall performance of the IgG serology was satisfactory. Our data are consistent with those obtained by Souza et al. when testing the recombinant forms of LJM11 and LJM17 from *Lu*. *longipalpis* in a large cohort of 1077 individuals randomly selected from an area of visceral leishmaniasis [19]. The effectiveness of using the combined salivary proteins in the prediction of anti-SGE antibody positivity has been assessed using ROC curves that evidenced AUC of approximately 0.8, a result similar with the one obtained herein.

In an attempt to improve the performance of the IgG ELISA, we tested whether detecting IgG subclasses and/or IgE antibodies against rPpSP32 would improve the serology performance. However, none has performed better than IgG rPpSP32 serology. Importantly, all tested sera that exhibited anti-SGE antibodies but that were negative for anti-SP32 antibodies by ELISA reacted against rPpSP32 using the Western blot technique for total IgG anti- rPpSP32 demonstrating a higher sensitivity compared to ELISA, a result consistent with previous data for antibodies against total SGE [38]. This suggests that a commercial strip test could be developed to measure the antibody response towards rPpSP32 by a finger prick capillary sampling method and the use of a simple auto-reactive strip to which rPpSP32 would be fixed. This technique would be particularly applicable on the ground in rural zones or for large-scale epidemiological studies and should provide a useful tool to measure vector-exposure on a large scale. Such technology, using a specific recombinant protein from *L*. *donovani*, has proven to be a rapid, efficient and easy test in serodiagnosis of visceral leishmaniasis [39].

Altogether, our results show that PpSP32 is a suitable marker of exposure to *P*. *papatasi* bites. However, recombinant molecules that could be used in serology should not react with antibodies towards salivary proteins of other sympatric sand fly species. Homologues of PpSP32 are, indeed, present in at least four other sand fly species (*P*. *argentipes*, *P*. *ariasi*, *P*. *perniciosus and Lu*. *longipalpis*) [29]. Among them, only *P*. *perniciosus* is prevalent in Tunisia [26]. In order to test the specificity of our serological test, we have previously tested sera from people living in one area where *P*. *perniciosus* is highly predominant and where *P*. *papatasi* is absent. None of them reacted against rPpSP32 suggesting the specificity of our serological test towards the SP32 from *P*. *papatasi* [24]. Herein, we have confirmed the absence of cross-reactivity between *P*. *papatasi* SP32 and *P*. *perniciosus* salivary antigens. Indeed, sera obtained from dogs that were exposed to *P*. *perniciosus* saliva through bites did not react against *P*. *papatasi* SP32. Recently, a homologue of PpSP32 (PsSP44 (HM569368)) has been described in another *Phlebotomus* species, *P*. *sergenti*, subgenus *Paraphlebotomus* [30]. The probable role of this rare vector (less than 1% of sand flies in arid regions of Tunisia) [26] in the transmission of *L*. *killicki* in Southwestern Tunisia has recently been proposed [40]. Since the reactivity of anti-*P*. *sergenti* antibodies with *P*. *papatasi* SGE is well documented [14], a possible cross-reactivity between PsSP44 and PpSP32 could not be excluded and should be studied. Nonetheless, for Tunisia and for practical purposes, the rarity of *P*. *sergenti* and the fact that antibodies to rPpSp32 will not likely recognize the SP32-like protein from *P*. *perniciosus* render rPpSP32 a suitable marker to evaluate *P*. *papatasi* exposure. To improve rPpSP32 as biomarker of *P*. *papatasi* exposure and to prevent possible cross-reactivity with other Sp32-like protein from sand fly species evolutionarily close to *P*. *papatasi* such *P*. *sergenti*, specific immunodominant epitopes could be designed from the rPpSP32 sequence using bioinformatics or epitope mapping approaches. By performing multiple sequence alignment of the Sp32-like proteins from different sand fly species, many of whose transcriptomes are available [30], we can exclude select cross reactive epitopes from PpSP32. A similar approach has been successfully implemented in salivary proteins from Anopheles mosquitoes [41]. The only drawback of this approach would be the possible exclusion of conformational epitopes.

The use of insect salivary proteins as markers of vector exposure is an area of research that is expanding to encompassing various vectors of disease including sand flies. The immunogenic nature of some of the salivary proteins present in vectors of disease makes them ideal candidates as tools to develop practical biomarkers of vector exposure. In this work, we have validated the use of a salivary protein that is highly immunogenic in humans, named rPpSP32, as a suitable biomarker to evaluate the exposure to *P*. *papatasi* sand flies in humans. As was achieved for other vectors of disease, rPpSP32 has the potential to be used in epidemiological studies to assess the risk of contracting vector borne-diseases, in this case ZCL. In our cohort, the number of new ZCL cases recorded during the two year follow-up was, however, insufficient to perform statistical tests to substantiate this claim. Yet, rPpSp32 could be used to practically monitor the spatial distribution of *P*. *papatasi* sand flies in conjunction with the study of reservoirs and entomological surveys. Importantly, rPpSP32 can be useful to assess the value of vector control measures. The success of such an approach has been demonstrated for biomarkers of vector saliva for assessment of the efficacy of insecticide impregnated nets [20, 42].
